# Evaluation of a radiomics nomogram derived from Fluoride-18 PSMA-1007 PET/CT for risk stratification in newly diagnosed prostate cancer

**DOI:** 10.3389/fonc.2022.1018833

**Published:** 2022-11-15

**Authors:** Zhuonan Wang, Yunxuan Li, Anqi Zheng, Jungang Gao, Wang Yuan, Cong Shen, Lu Bai, Xiaoyi Duan

**Affiliations:** PET/CT Center, The First Affiliated Hospital of Xi’an Jiaotong University, Xi’an, China

**Keywords:** prostate cancer, 18F-PSMA-1007 PET/CT, risk stratification, radiomics, nomogram

## Abstract

**Objective:**

The aim of this study was to evaluate the performance of Fluoride-18 (^18^F)-PSMA-1007-PET/CT radiomics for the tumor malignancy and clinical risk stratification in primary prostate cancer (PCa).

**Materials and Methods:**

A total of 161 pathological proven PCa patients in a single center were retrospectively analyzed. Prostate-specific antigen (PSA), Gleason Score (GS) and PET/CT indexes (SUVmin, SUVmax, and SUVmean) were compared according to risk stratification. Radiomics features were extracted from PCa ^18^F-PSMA-1007-PET/CT imaging. The radiomics score integrating all selected parameters and clinicopathologic characteristics was used to construct a binary logistic regression and nomogram classifier. Predictors contained in the individualized prediction nomogram included radiomics score, PSA level and metastasis status.

**Results:**

The radiomics signature, which consisted of 30 selected features, was significantly associated with PSA level and Gleason score (P < 0.001 for both primary and validation cohorts). Predictors contained in the individualized prediction nomogram included radiomics score, PSA level and metastasis status. The model showed good discrimination with an area under the ROC curve of 0.719 for the GS. Combined clinical-radiomic score nomogram had a similar benefit to utilizing the PET/CT radiomic features alone for GS discrimination.

**Conclusion:**

The ^18^F-PSMA-1007-PET/CT radiomics signature can be used to facilitate preoperative individualized prediction of GS; incorporating the radiomics signature, PSA level, and metastasis status had similar benefits to those of utilizing the PET/CT radiomics features alone.

## Introduction

Prostate cancer (PCa) is one of the leading causes of death in men worldwide, and its biological behavior is highly heterogeneous, directly affecting patient prognosis and treatment options (1–4). During prostate screening, elevated prostate-specific antigen (PSA) levels indicate an increased risk of PCa; however, PSA is organ-specific but not tumor-specific, and the specificity of PSA in reflecting disease severity remains debatable (5). Using PSA-level as the only indicator for PCa risk stratification discrimination lacks specificity, causing a large number of unnecessary prostate biopsies (6–9). In addition, according to guidelines, PCa patients with PSA ≥20 ng/mL and/or Gleason score (GS) ≥8 are at high risk; the probability of metastasis and mortality is significantly higher and such patients may not be suitable for active surveillance programs, radical prostatectomy, or radiotherapy treatment (5, 10–12). In addition, elderly patients with severe comorbidities or undergoing anticoagulation therapy may not be optimal candidates for biopsies and may experience adverse effects, leading to higher healthcare costs (7). Therefore, finding an accurate and objective imaging biomarker to assess tumor phenotype using a non-invasive approach will play a crucial role in PCa.

Prostate-specific membrane antigen (PSMA) is a type II transmembrane glycoprotein overexpressed in PCa cells, and its expression correlates with the degree of malignancy (13, 14). The ^18^F-PSMA-1007 PET/CT is a reliable PSMA-targeting positron emission tomography (PET)-tracer. Because of better spatial resolution images compared with other PSMA-targeting radiotracers that can be obtained and non-urinary route of excretion that reduces urinary clearance, this approach has great potential to facilitate the detection of primary PCa (15–18). Although previous studies have investigated the positive correlation between ^18^F-PSMA tracer uptake and GS, using the PSMA signal and semi-quantitative PET/CT parameters to distinguish tumor malignancy remains uncertain (19, 20). Radiomics may provide quantitative and objective support for decisions surrounding cancer detection, and extracting quantitative features may identify imaging biomarkers to predict treatment outcomes and non-invasively characterize tumor biology (21). Prior studies have evaluated PET image features for lymph node metastasis detection in patients with primary PCa (22, 23), and whether radiomics features derived from ^18^F-PSMA-1007 PET/CT imaging in primary PCa may identify patients with tumor malignancy is not known.

The aim of this study was to retrospectively evaluate the role of radiomics derived imaging features from ^18^F-PSMA-1007 PET/CT in tumor malignancy discrimination and risk stratification. In addition, we investigated the feasibility of radiomics features combined with clinical parameter validation for GS prediction.

## Materials and methods

### Patients

**Figure 1:**
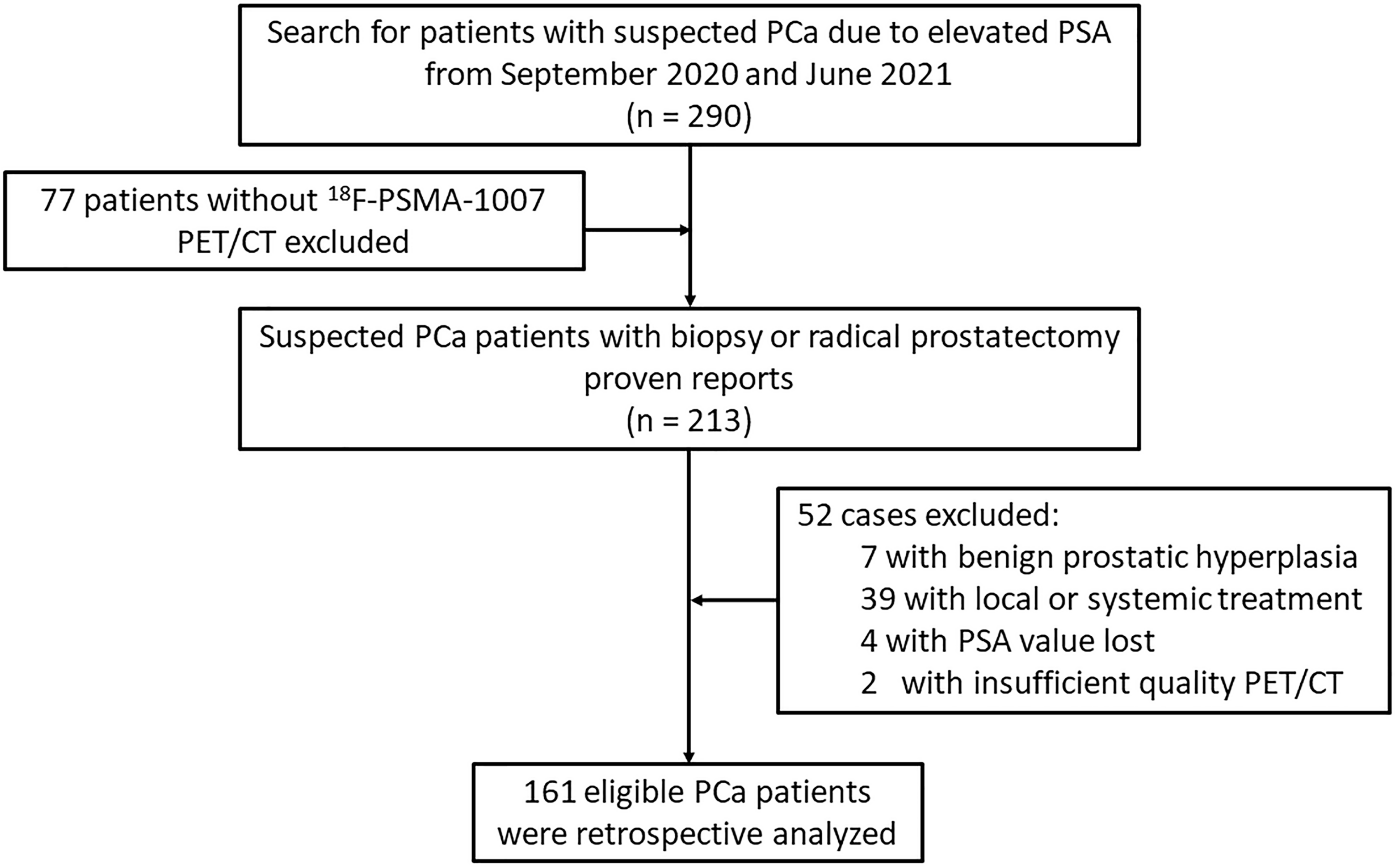
Flowchart of the prostate cancer patient’s cohort.

Ethical approval was obtained from the Institutional Review Board and was conducted in accordance with the Declaration of Helsinki. Informed consent was obtained from all individual participants included in the study. This study enrolled 290 consecutive patients with suspected primary PCa between September 2020 and June 2021 in a single center. The GS were obtained from 12 to 14 core systematic transrectal ultrasonography guided prostate biopsy (TRUS) at initial evaluation, and if present, results of radical prostatectomy were documented additionally. A higher-grade Gleason score were then selected for analysis. The interval between biopsy/surgical results and ^18^F-PSMA-1007 PET/CT scan were not exceed one week. All participants included in the data analysis were evaluated using ^18^F-PSMA-1007 PET/CT and had PSA values measured within 4 weeks prior to the imaging scan. Diagnosis of PCa proven through histological examination served as a reference for PET imaging analyses (4, 24). Patients were excluded from the analysis if they 1) had received local or systemic treatment, 2) lacked histological examination proven diagnosis or PSA value, and 3) had incomplete imaging data. Finally, 161 eligible patients were retrospectively analyzed (**Figure 1**). All patients were dichotomized for machine learning based classification using the following criteria: PSA, <20 vs. ≥20 ng/mL; GS, <8 vs. ≥8; and presence of any metastasis, N0 and M0 vs. N1 and/or M1. Of note, the “any metastasis” outcome is an expansion of patients with lymph node involvement to include patients with distant metastases (25).

### ^18^F-PSMA-1007 and image acquisition

All ^18^F-PSMA-1007 PET/CT data were acquired using a PET/CT scanner (Gemini 64TF, Philips, Netherlands) at a single location. Radiolabeling was performed using a fully automated radiopharmaceutical synthesis device based on a modular concept (MINItrace, GE Healthcare, USA). Over 99% radiochemical purification yield ^18^F-PSMA was obtained and examined using both radio-thin layer chromatography and high performance liquid chromatography. Patients received intravenous injection of ^18^F-PSMA-1007 (3.7 MBq/kg body weight) and underwent PET and CT scans 90 min after injection. Low-dose CT scans from the head to the proximal thigh (pitch 0.8 mm, 60 mAs, 120 kV [peak], tube single turn rotation time 1.0 s, and 5-mm slice thickness) and for PET attenuation were acquired (pitch 0.8 mm, automatic mAs, 120 kV [peak], and 512 × 512 matrix). PET data sets were reconstructed using time-of-flight with three iterations. Whole-body PET scans were performed in three-dimensional mode (emission time: 90 s per bed position, scanned for a total of 7–10 beds) as our prior study (4).

### Tumor delineation

**Figure 2:**
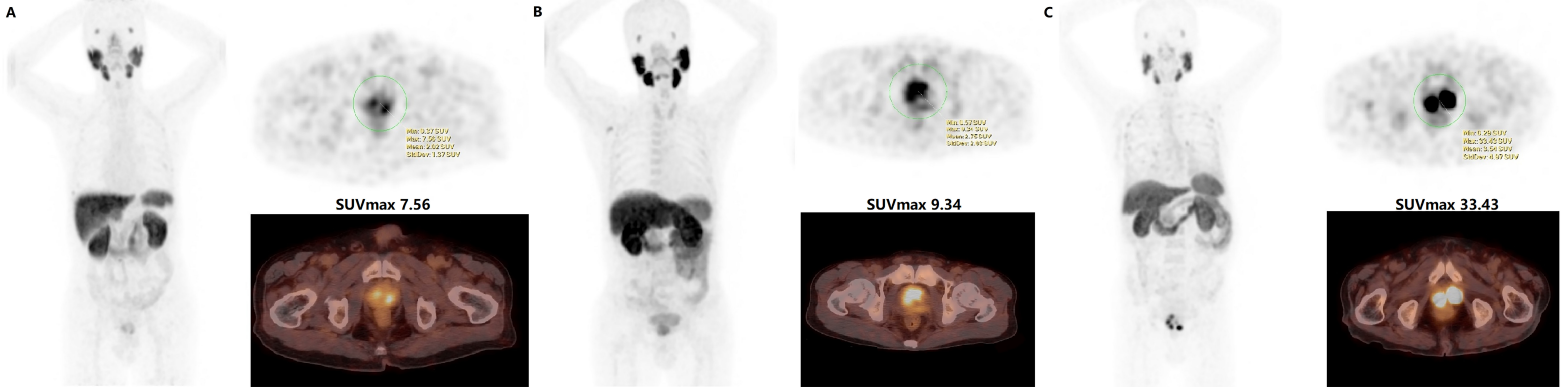
**(A)** Low - grade (Gleason 6, ISUP 1); **(B)** Intermediate - grade (Gleason 7, ISUP 3); **(C)** High - grade (Gleason 9, ISUP 5) prostate cancer with SUVmax 7.56, SUVmax 9.34 and SUVmax 33.43 on ^18^F-PSMA-1007 PET/CT, respectively. Whole-body maximum intensity projection (MIP) image (left); axial PET image (right up) and axial fused image (right bottom) for each patient.

Two experienced nuclear medicine specialists jointly interpreted all ^18^F-PSMA-1007 PET/CT scans and performed a comprehensive analysis of the available clinical data. A consensus was reached through discussion when the conclusions of the two specialists were discordant. Intraprostatic tumor localization on ^18^F-PSMA-1007 PET/CT was adapted to lesions with focally increased uptake corresponding to the tumor site verified during Transrectal Ultrasonography biopsy or radical prostatectomy (**Figure 2**) (26). The initial evaluation was to assess whether the primary malignancy tumor was visually distinguishable from surrounding prostate tissue. The location of the primary tumor on the PET image should correspond to the area of the PCa mass that was systematically 12-14 needles biopsy. The identified metastases were consistent with the pathological tracer accumulation of PCa lesions (5, 27, 28). Tracer positive lesions were also composite validated based on other imaging approaches, disease management, and PSA measurements.

### Tumor delineation image segmentation

Image segmentation and extraction were conducted using ITK-SNAP Version 3.8 (29). Two experienced nuclear medicine specialists jointly interpreted whether the prostate tumor invaded the surrounding tissue. If the lesion was confined to the local prostate, the prostate tissue was extracted using spherical delineation or slice by slice (6-12 slices); if there was an infiltration of the bladder, combined with CT imaging, the region of interest was manually drawn slice by slice on the PET image, and avoiding surrounding invaded tissue. After segmenting the prostate tissue, we first extracted the maximum standardized uptake value (SUVmax), and then used 40% of the SUVmax as the lower threshold to achieve extract prostate tumor tissue.

### Radiomics feature extraction

Radiomics feature extraction was implemented using a Philips Radiomics Tool (Philips Healthcare, China), and the core feature calculation was based on pyRadiomics (30). For each VOI, a total of 944 three-dimensional (3D) radiomic features, including direct features, wavelet transform features, logarithm transform features, and gradient filtered features, were extracted (types, introduction of extracted features, and number of each type are shown in **Supplementary Materials**); details can be found in pyradiomics.readthedocs.io/en/latest/features.html. First order statistics describe the distribution of voxel intensities within the image region defined by the mask through commonly used and basic metrics. In the group of shape features we included descriptors of the three-dimensional size and shape of the ROI. All radiomics features were normalized to the z-score. Pearson’s rank correlation coefficient analysis of each pair of features was performed, and details can be found in the **Supplementary Materials**.

### Statistical analysis

Statistical analysis was conducted using R software (version 4.0.2; http://www.Rproject.org) and SPSS 13.0 Version. The packages in R used in this study are reported in the **Supplementary Materials**. Patient data are shown as median, mean, standard deviation, range, or percentage where applicable. The reported statistical significance levels were all two-sided, with statistical significance set at 0.05. The Wilcoxon–Mann–Whitney U test was used to test clinical subgroups (PSA <20 vs. ≥20 ng/mL, GS ≥8 vs. GS <8, and metastasis status) and PET index differences (including minimum SUV [SUVmin], SUVmax, and mean SUV [SUVmean]).

The least absolute shrinkage and selection operator (LASSO) method, which is suitable for the regression of high dimensional data, was used to select the most useful predictive features (31). The performance of these models in predicting malignancy was first evaluated in the training cohort and then in a testing cohort by plotting a receiver operating characteristic (ROC) curve and calculating the area under the curve (AUC). The corresponding sensitivity, specificity, negative predictive value (NPV), and positive predictive value (PPV) were calculated. Calibration curves were plotted to assess the calibration of the radiomics signatures.

A radiomics score was calculated for each patient using a linear combination of selected features that were weighted by their respective coefficients (32). To predict the GS, we developed a radiomics nomogram using multivariable logistic regression analysis (PSA value and metastasis status) and radiomics score. Moreover, the Hosmer–Leme show test was used to quantify the performance of the radiomics nomogram. Decision curve analysis was conducted to determine the clinical usefulness of the radiomics nomogram by quantifying the net benefits at different threshold probabilities in the validation dataset. The decision curve was also plotted for the model after the addition of PSA and metastatic status.

## Results

### Clinical characteristics

A total of 161 patients with biopsy (139 patients) or radical prostatectomy (22 patients) proven PCa were included. Among the patients who underwent radical prostatectomy, 5 patients had inconsistent GS results with the biopsy. The demographic information and clinical characteristics of the participants are summarized in **Table 1**. Among these patients, 82 patients had PSA levels <20 ng/mL and 79 patients had PSA levels higher than ≥20 ng/mL. The number of low-intermediate-grade patients was 68 (GS <8) and the number of high-grade patients was 93 (GS ≥8). According to metastatic findings on ^18^F-PSMA-1007 PET/CT, 86 patients had no metastasis and 75 patients had metastasis. Semi-quantitative analysis of ^18^F-PSMA-1007 PET/CT was performed for all patients. Comparing the subgroups with PSA ≥20 ng/mL and <20 ng/mL, and GS ≥8 and GS <8, the PET/CT semi-quantitative variables (SUVmin, SUVmax, and SUVmean) of the former group were all significantly higher than those of the latter group; no difference was found in metastasis status (**Table 2**).

**Table 1:** Demographic and clinical characteristics of the primary prostate cancer patients.

Characteristic			Value		
Age (range)			73 (42–95)		
Mean ± SD			71.44 ± 8.87		
PSA (ng/mL) Mean ± SD			18.59 (0.17–3022) 91.57 ± 293.14		
Non-metastatic patients (%)			86 (53.42%)		
Metastatic patients (%)			75 (46.58%)		
cN1M0			20 (12.42%)		
cM1a			8 (4.97%)		
cM1b			40 (24.84%)		
cM1c			7 (4.35%)		
Gleason score		ISUP		NCCN Risk Stratification	
6	14 (8.7%)	1	14 (8.7%)	Very low	0 (0%)
7	54 (33.5%)	2	8(5.0%)	Low	3 (1.86%)
8	29 (18.0%)	3	46(28.5%)	Intermediate	58 (36.02%)
9	64 (39.8%)	4	29 (18.0%)	High	50 (31.06%)
10	0 (0%)	5	64 (39.8%)	Very High	50 (31.06%)

SD, standard deviation; PSA, prostate-specific antigen. Low-intermediate risk: PSA <10 ng/mL and Gleason = 6, or PSA 10–20 ng/mL OR Gleason = 7. High risk: PSA ≥20 ng/ml OR Gleason ≥8. ISUP: International Society of Urological Pathology. N1: Regional lymph node metastasis, M0 No distant metastasis, M1a Non-regional lymph node(s), M1b Bone(s), M1c Other site(s).

**Table 2:** 18F-PSMA-1007 PET/CT parameters for different PSA, Gleason score, and metastasis status subgroups.

Categorical variable	PSA <20 ng/mL n = 82	PSA ≥20 ng/mL n = 79	Sig	GS <8 n = 68	GS ≥8 n = 93	Sig	Non-metastasis n = 86	Metastasis n = 75	Sig
SUVmin Mean ± SD (range)	5.80 ± 5.47 1.36-28.87	8.88 ± 6.43 0.60-37.95	*P = 0.001*	5.69 ± 4.39 1.41-15.18	8.50 ± 6.94 0.6-37.95	*P = 0.002*	8.15 ± 6.83 1.41-37.95	6.35 ± 5.12 0.60-24.50	*P = 0.058*
SUVmax Mean ± SD (range)	20.31 ± 16.18 3.89-76.86	28.88 ± 7.76 3.60-101.89	*P = 0.002*	20.63 ± 12.97 3.60-57.77	27.35 ± 19.71 3.89-101.89	*P = 0.01*	26.23 ± 19.18 4.38-101.89	22.54 ± 15.15 3.60-62.95	*P = 0.181*
SUVmean Mean ± SD (range)	9.31 ± 8.10 1.84–41.55	13.85 ± 9.61 1.80–57.85	*P = 0.001*	9.18 ± 6.28 1.80–31.43	13.26 ± 10.45 1.84-57.85	*P = 0.002*	12.71 ± 10.09 2.23-57.85	10.19 ± 7.75 1.80-37.48	*P = 0.075*

### Feature selection and radiomics signature building

**Figure 3:**
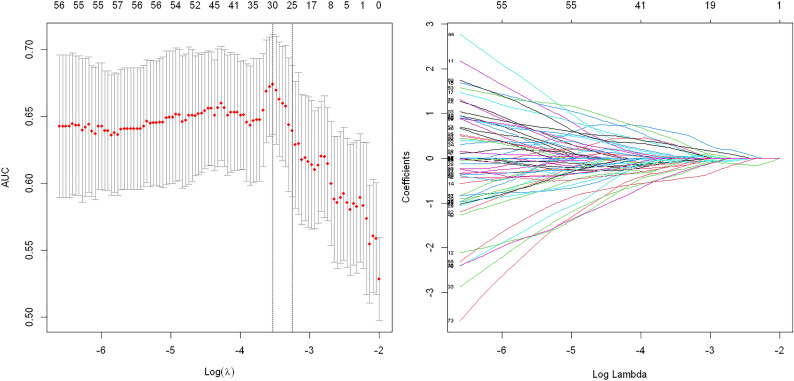
Texture feature selection using the least absolute shrinkage and selection operator (LASSO) binary logistic regression model for Gleason score subgroups. Tuning parameter: dotted vertical lines were drawn at the optimal values using the minimum criteria and (λ) standard error of the minimum criteria. The area under the receiver operating characteristic curve (AUC) was plotted against log (λ). The value of 0.6893 was chosen, and the optimal (λ) resulted in 30 nonzero coefficients.

A total of 944 features were reduced to 30 potential predictors (30:1 ratio; **Figures 3**) and were features with nonzero coefficients in the LASSO logistic regression model penalty *via* minimum criteria for the GS sub-group.

### Radiomics features for discrimination of high Gleason score vs. low Gleason score and high PSA level vs. low PSA level

**Figure 4:**
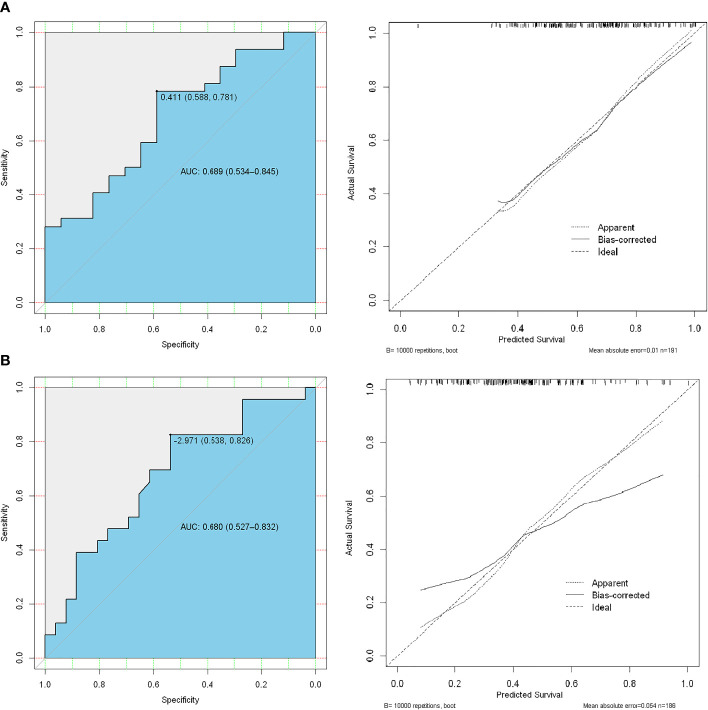
ROC analysis of the prediction model using high-risk Gleason score (≥8) vs. low-intermediate-risk Gleason score (<8) **(A)**, the first line) and high-risk PSA level (≥20 ng/mL) vs. low-risk (<20 ng/mL) PSA level **(B)**, the second line).

There was a significant difference in the radiomics score between low–intermediate-grade and high-grade patients in both the training (112 patients) and test (49 patients) cohorts (P < 0.01); the ROC AUC was 0.689 (P < 0.01) for the test cohort with a sensitivity of 58.8%, specificity of 78.1%, PPV of 77.4%, and NPV of 55.6% (**Figure 4A**). We also found a significant difference in radiomics score between the low and high PSA level subgroups in both the training and test cohorts (P < 0.01); the ROC AUC value was 0.680 (P < 0.01) for the test cohort with a sensitivity of 60.6%, specificity of 62.5%, PPV of 76.9%, and NPV of 43.5% (**Figure 4B**).

### Development of an individualized prediction model

**Figure 5:**
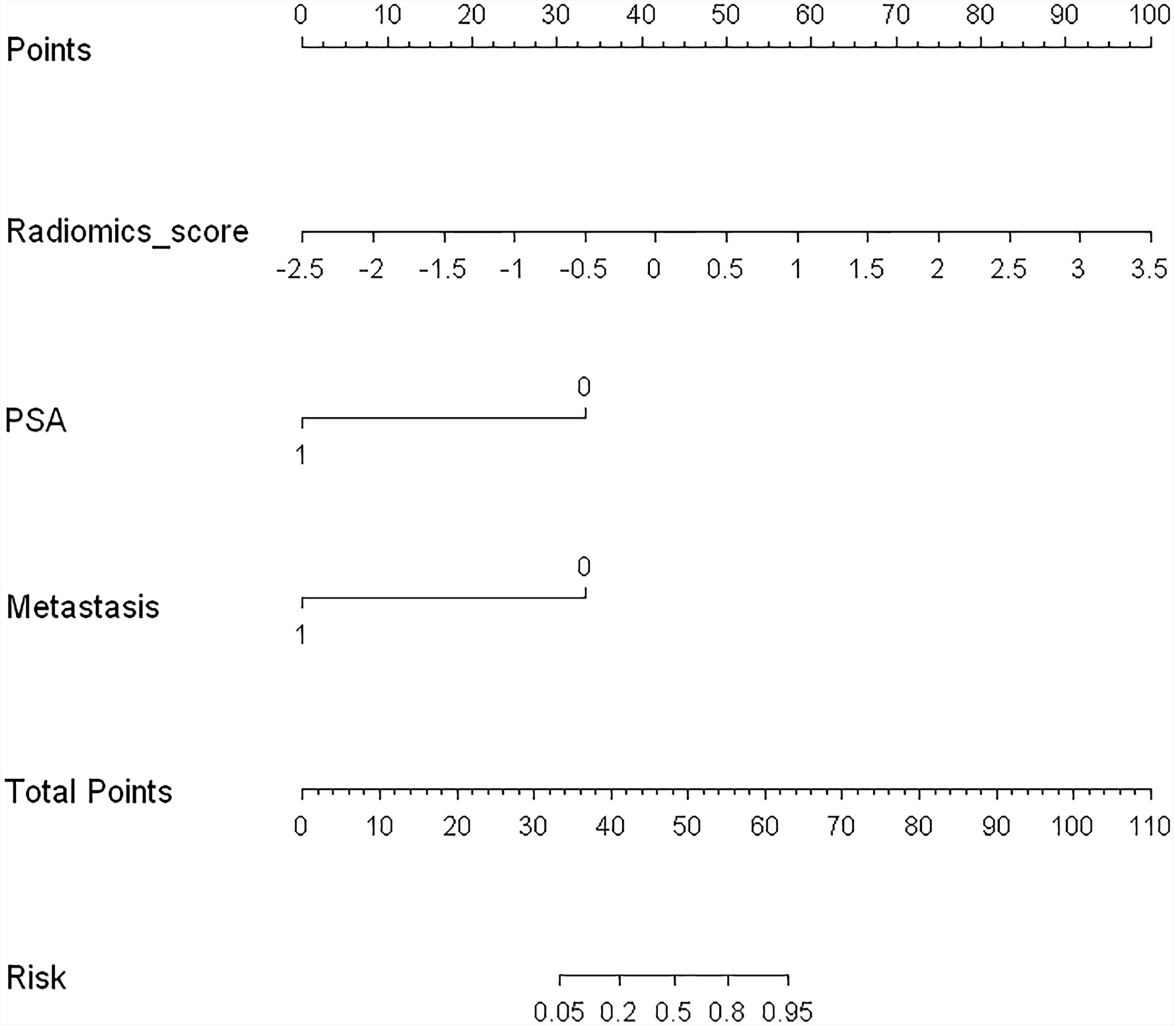
Developed radiomics nomogram. The radiomics nomogram was developed in the training cohort, with the radiomics signature, PSA level, and PET/CT-reported metastatic status incorporated.

Logistic regression analysis identified the radiomics signature, PSA level, and PET/CT reported metastatic status as independent predictors. A model that incorporated the above independent predictors was developed and presented as a nomogram (**Figure 5**).

### ROC analysis and clinical application

**Figure 6:**
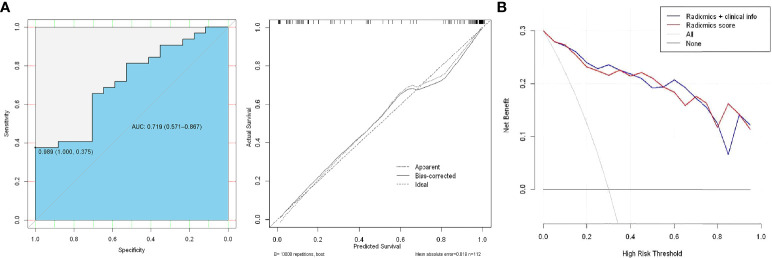
**(A)** ROC analysis of the prediction model using the radiomic nomogram and decision curve analysis of the radiomics model. The y-axis represents net benefit. **(B)** The red and blue lines represent the radiomic nomogram and radiomic nomogram and clinical combination, respectively.

In the ROC analysis, the AUC was 0.719 (95% CI: 0.571–0.867, P < 0.01) for the test cohort with a sensitivity of 47.1%, specificity of 81.3%, PPV of 74.3%, and NPV of 43.5% (**Figures 6A**). The use of the combined clinical-radiomic nomogram had a similar benefit as that of using the GS radiomic features alone (**Figure 6B**).

## Discussion

The ^18^F-PSMA tracer PET/CT has been reported to be more favorable for primary tumors and play an important role in pre-treatment setting and high-risk PCa monitoring. However, because the visual method is subjective, it is prone to misinterpretation, and there may be abnormal uptake of PSMA both in PCa lesions and hyperplastic foci, which makes the diagnosis of malignant prostate disease challenging. The PSMA PET based radiomics to characterize PCa aggressiveness on histopathology and whole-body scanning has the potential to make PSMA PET/CT a one-stop shop for individualized PCa management and prognosis assessment. Previous radiomics studies have mainly focused on multiparameter magnetic resonance imaging (MRI) to distinguish between benign and malignant prostate neoplasms or different levels of malignancy (33, 34). Radiomics promises the benefit of automation, which can reduce human error and prevent unnecessary biopsies associated with misdiagnosis. However, the ability of ^18^F-PSMA-1007 PET/CT radiomic features to predict the GS of PCa to estimate the aggressiveness of a tumor remain largely unexplored.

Several studies have highlighted the high sensitivity and application value of PSMA PET/CT in the risk stratification and metastatic findings of primary PCa (3, 26, 35). Our study confirmed that the ^18^F-PSMA-1007 PET/CT variables, SUVmin, SUVmax, and SUVmean, can be used as semi-quantitative “imaging biomarkers” for both PSA levels (<20 vs. ≥20 ng/mL) and pathological malignancy (GS <8 vs. ≥8) discrimination among patients with PCa. No statistically significant differences were found between the metastatic and non-metastatic subgroups. The current study further found that ^18^F-PSMA-1007 PET/CT radiomic features are promising markers for the noninvasive discrimination of low-intermediate and high pathological risk, and different PSA serum values (<20 vs. ≥20 ng/mL) in patients with PCa. Prior multi-parameter MRI radiomics studies have shown machine learning prediction models with the capacity to discriminate between GS of ≥8 and <8 with an AUC of 0.72 (33). Our study had similar findings, we included patients with GS from 6 to 9 and used ^18^F-PSMA-1007 PET/CT radiomic features as predictors, which had stable performance in the validation group with an AUC of 0.689, sensitivity of 58.8%, and specificity of 78.1%. The performance in our study could be explained by the finding that substantial changes are more likely between PCa with GS of 6 and 7, and the intermediate-risk group are more likely to show statistically significant differences between PCa with a GS of 7 and GS ≥8 with higher AUC values (33, 36). The prior review has mentioned that bioptic GS has a significant impact on clinical management as it defines the patient’s risk group, and directly influences the duration of androgen deprivation therapy or the dose to the prostate during radiation therapy (37, 38).

Our study integrated the radiomics score, PSA category level, and metastatic status for GS discrimination validation. With an AUC of 0.719, the radiomics nomogram had a higher specificity (81.3%) than the radiomics features alone (78.1%). In the assessment of prostate malignancy and prognosis, it is possible to establish an effective diagnosis of pathological malignancy in the early stage. Avoiding non tumor specific PSA and subjective PET/CT visual validation errors plays a crucial role in determining clinical treatment options. Therefore, to justify its clinical usefulness, we assessed whether the radiomics features combined with PSA levels and metastasis findings in a nomogram would improve patient outcomes. The decision curve analysis revealed that the benefit of the nomogram distinguishing GS may be similar to that of the radiomics signatures. Thus, to reduce unnecessary prostate biopsies for tumor malignancy prediction, radiomics signatures provide a more objective and independent prediction scheme.Prior study found that the detection rate of distant metastasis for ^18^F-PSMA PET/CT was higher between the PSA ≥ 30ng/mL and PSA ≥ 20ng/mL cohorts. The PET/CT parameter SUVmax difference between primary tumors and metastatic lesions in metastatic PCa patients with a higher prediction PSA level (i.e., > 29.01) (4). Therefore, benefiting populations in a nomogram based on PSA, metastatic status, and radiomics score may be more likely to be represented in patients with higher PSA.

Our study has several limitations. The results require external validation on a larger scale prior to broader clinical applications. Future work in this area should consider combining PET/CT imaging findings with pathological results regarding metastatic lesions to improve overall diagnostic accuracy. The manual segmentation method described is potentially measurement error, adopting automatic separation in the future will help improve accuracy. Our ongoing research has incorporated CT radiomics features and another segmentation method into the analysis for improving diagnostic performance.

In conclusion, using ^18^F-PSMA-1007 PET/CT radiomics features to distinguish the degree of malignancy of the primary tumor might be helpful. Incorporating the radiomics signature, PSA level, and metastasis status had similar benefits to those of utilizing the PET/CT radiomics features alone.

## Data availability statement

The original contributions presented in the study are included in the article/**Supplementary Material**. Further inquiries can be directed to the corresponding author.

## Ethics statement

The studies involving human participants were reviewed and approved by The First Affiliated Hospital of Xi’an Jiaotong University institutional review board (No. 2019LSYZD-J1-H). The patients/participants provided their written informed consent to participate in this study.

## Author contributions

ZW drafted the manuscript, contributed to the conception and design, analysis and interpretation of data. YL and AZ contributed to the analysis and interpretation of data. JG, WY, CS and LB contributed to acquisition of data. XD contributed to the revision of the manuscript critically for important intellectual content. All authors contributed to the article and approved the submitted version.

## Funding

This research was supported by the National Natural Science Foundation of China (Nos. 82001772), the Natural Science Foundation of Shaanxi Province, China (2021SF-062, 2020JZ-38), and the New Medical and Technology of the First Affiliated Hospital of Xi’an Jiaotong University (XJYFY-2019J1).

## Acknowledgments

We want to express our gratitude to Dr. Dong Han and Ph.D. Chengyu Ding for the contribution in performing this study.

## Conflict of interest

The authors declare that the research was conducted in the absence of any commercial or financial relationships that could be construed as a potential conflict of interest.

## Publisher’s note

All claims expressed in this article are solely those of the authors and do not necessarily represent those of their affiliated organizations, or those of the publisher, the editors and the reviewers. Any product that may be evaluated in this article, or claim that may be made by its manufacturer, is not guaranteed or endorsed by the publisher.
