# Supplementary material for: Evaluation of a radiomics nomogram derived from Fluoride-18 PSMA-1007 PET/CT for risk stratification in newly diagnosed prostate cancer
Source: Front Oncol. 2022 Nov 15;12:1018833. doi: 10.3389/fonc.2022.1018833 (PMC9705356; doi:10.3389/fonc.2022.1018833)
Supplement: Supplementary file 1 [file DataSheet_1.docx]

Supplementary Material

# Supplementary Data

**Radiomics Feature Extraction**

Direct features :107, Including first-order statistics features, shape-based features, gray level co-occurrence matrix (GLCM) features, gray level size zone matrix (GLSZM) features, gray level run length matrix (GLRLM) features, neigbouring gray tone difference matrix (NGTDM) features and gray level dependence matrix (GLDM) features

Wavelet transform features: 744, Information about the frequency of similar SIs and describes the wavelet transform of the pixels in the ROI

Logarithm transform features: 93, Information about the frequency of similar SIs and describes the logarithm transform of the pixels in the region of interest (ROI)

Gradient transform features: 93, Information about the frequency of similar SIs and describes the gradient transform of the pixels in the ROI

First-order statistics describe the distribution of voxel intensities within the image region defined by the mask through commonly used and basic metrics.

In the group of shape features we included descriptors of the three-dimensional size and shape of the ROI.

A Gray Level Co-occurrence Matrix (GLCM) describes the second-order joint probability function of an image region constrained by the mask. For example, autocorrelation is a measure of the magnitude of the fineness and coarseness of texture.

A Gray Level Size Zone (GLSZM) quantifies gray level zones in an image.

A Gray Level Run Length Matrix (GLRLM) quantifies gray level runs, which are defined as the length in number of pixels, of consecutive pixels that have the same gray level value.

A Neighbouring Gray Tone Difference Matrix quantifies the difference between a gray value and the average gray value of its neighbours within given distance.

A Gray Level Dependence Matrix (GLDM) quantifies gray level dependencies in an image {van Griethuysen, 2017 #6327}.

**Data** **Supplement**

Lasso binary logistic regression was done using the 'glmnet' package.

Intraclass correlation coefficient (ICC) calculation was performed with the 'irr' package.

Multivariate binary logistic regression, nomograms and calibration plots were performed the 'rms' package.

Decision curve analysis was performed with the 'rmda' package.

ROC analysis was performed with the 'pROC' package.

Hosmer-Lemeshow test was performed with the 'ResourceSelection' package.

**Feature Selection and Radiomics Signature Building**

First, all radiomics features were normalized to the z-score, as shown below:


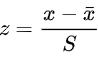


where:

{\displaystyle z={x-{\bar {x}} \over S}}

{\displaystyle {\bar {x}}}{\bar {x}} is the mean of the sample.

S is the standard deviation of the sample. [https://en.wikipedia.org/wiki/Standard_score]

Next, Pearson's rank correlation coefficient analysis between each pair of features was performed. A Pair of features with a coefficient greater than an absolute value of 0.9 was highlighted, and then one of these two features, the correlation between which and the label was more strong, was selected (Then we had 183 features left). We used the least absolute shrinkage and selection operator (LASSO) algorithm for dimensionality reduction [2-3]. A radiomics score (Rad-score) calculated for each patient via a linear combination of selected features that were weighted by their respective coefficients [4].

[1] van Griethuysen JJM, Fedorov A, Parmar C, Hosny A, Aucoin N, Narayan V, Beets-Tan RGH, Fillion-Robin JC, Pieper S, Aerts H: Computational Radiomics System to Decode the Radiographic Phenotype. *Cancer Res* 2017, **77**(21):e104-e107.

[2] Huang YQ, Liang CH, He L, Tian J, Liang CS, Chen X, Ma ZL, Liu ZY: Development and Validation of a Radiomics Nomogram for Preoperative Prediction of Lymph Node Metastasis in Colorectal Cancer. J Clin Oncol 2016, 34(18):2157-2164.

[3] Sauerbrei W, Royston P, Binder H: Selection of important variables and determination of functional form for continuous predictors in multivariable model building. Stat Med 2007, 26(30):5512-5528.

[4] Zheng BH, Liu LZ, Zhang ZZ, Shi JY, Dong LQ, Tian LY, Ding ZB, Ji Y, Rao SX, Zhou J et al: Radiomics score: a potential prognostic imaging feature for postoperative survival of solitary HCC patients. BMC Cancer 2018, 18(1):1148.
